# Impact of nurse-led self-management education on type 2 diabetes: a meta-analysis

**DOI:** 10.3389/fpubh.2025.1622988

**Published:** 2025-08-11

**Authors:** Jiabao Sun, Zhenwei Fan, Mengyuan Kou, Xuewei Wang, Zhongmin Yue, Min Zhang

**Affiliations:** School of Nursing, Beihua University, Jilin City, Jilin, China

**Keywords:** type 2 diabetes mellitus, diabetes self-management education, nurse-led, meta-analysis, self-efficacy

## Abstract

**Background:**

Diabetes Self-Management Education (DSME) is a cornerstone strategy for improving glycemic control, yet its clinical effectiveness is often limited by suboptimal adherence. The aim of this study is to evaluate through meta-analysis the impact of nurse-led DSME on glycemic control, lipid profiles, and self-efficacy in adults with type 2 diabetes mellitus (T2DM).

**Methods:**

Following PRISMA 2020 guidelines, we systematically searched PubMed, EMBASE, and Web of Science databases (up to February 28, 2025). Inclusion criteria comprised: randomized controlled trials (RCTs) comparing nurse-led DSME (≥3 structured sessions) vs. usual care or other non-nurse-led interventions. Risk of bias was assessed using Cochrane RoB 2.0. Effect sizes [mean difference (MD) or standardized mean difference (SMD)] were pooled using R meta package with random-effects models (*I*^2^ > 50%). Subgroup analyses and meta-regression were performed.

**Results:**

Eight RCTs (reporting HbA1c outcomes) were included. Meta-analysis demonstrated: (1) Glycemic control: nurse-led DSME significantly reduced HbA1c at 4–6 months (MD = −0.92, 95% CI: −1.44 to −0.41) and >6 months (MD = −0.54, 95% CI: −0.86 to −0.23; *p* < 0.05), but not at 0–3 months (MD = −0.22, 95% CI: −1.15 to 0.51). Fasting blood glucose (FBG) showed significant improvement (MD = −0.20, 95% CI: −0.36 to −0.03). (2) Self-efficacy: the intervention group demonstrated significantly enhanced self-efficacy (SMD = 1.48, 95% CI: 1.04–1.92). (3) Lipid profiles: high-density lipoprotein (HDL) increased significantly (MD = 0.27, 95% CI: 0.14–0.41), while total cholesterol (TC), triglycerides (TG), and low-density lipoprotein (LDL) showed no significant changes. (4) Considerable heterogeneity was observed (HbA1c: *I*^2^ = 87.8%; self-efficacy: *I*^2^ = 84.5%). Meta-regression suggested borderline significant influence of follow-up duration on effect size (*p* = 0.059). No significant publication bias was detected (Egger's test *p* = 0.116).

**Conclusion:**

Nurse-led DSME effectively improves long-term glycemic control and self-efficacy while elevating HDL levels in T2DM patients, though standardization of intervention protocols is needed to reduce heterogeneity. This study supports integrating nurse-led models into diabetes management guidelines and recommends future research focus on long-term follow-up and cost-effectiveness analyses.

**Systematic review registration:**

https://doi.org/10.37766/inplasy2025.7.0114, identifier: NPLASY202570114.

## 1 Introduction

Diabetes Self-Management Education (DSME)—a structured process that equips individuals with the knowledge, skills, and confidence required for effective self-care—is recognized as a cornerstone in the comprehensive management of type 2 diabetes mellitus (T2DM). It encompasses critical areas such as glycemic monitoring, medication adherence, dietary regulation, physical activity, and psychosocial support, all of which are essential to achieving long-term glycemic control and reducing diabetes-related complications (1). Despite the proven efficacy of DSME, its real-world impact is often constrained by suboptimal adherence. Evidence suggests that over 50% of individuals with T2DM demonstrate low engagement in self-management behaviors, and only a small fraction consistently adhere to core practices such as dietary modification and regular blood glucose monitoring (2). This challenge is particularly pressing given the escalating global burden of T2DM, which has emerged as a major public health concern. Recent epidemiological data project that the number of individuals affected could surpass 750 million within the next two decades (3). T2DM is a leading contributor to morbidity and mortality through its strong association with cardiovascular disease, nephropathy, and retinopathy. Clinically, the significance of glycemic control is underscored by findings that each 1% increase in HbA1c corresponds to nearly a 40% rise in both microvascular and macrovascular complication risks, as well as all-cause mortality (4).

While pharmacotherapy remains foundational, nearly 50% of patients fail to achieve glycemic targets (HbA1c < 7%), underscoring the need for effective adjunctive strategies (5, 6). DSME is a critical yet underutilized intervention, especially when delivered through nurse-led models that enhance accessibility, continuity of care, and patient-centered communication. A meta-analysis of 25 studies confirmed that DSME significantly improves HbA1c levels (7). However, its clinical translation is hindered by low patient adherence—over half struggle with consistent self-management, and only 8.33% sustain high-level behaviors such as dietary control and glucose monitoring (8).

Nurse-led DSME is a unique care model that leverages nurses' strengths in continuity of care, educational expertise, and cultural sensitivity. Unlike physician-led approaches, nurse-led continuity of care significantly enhances patient adherence (9). Although the effectiveness of nurse-led DSME is widely recognized, its specific contributions remain controversial. DSME is recognized as a foundational intervention for improving glycemic control, its real-world effectiveness is often constrained by inconsistent patient adherence. Among the included studies, adherence to DSME was variably reported and, when mentioned, was not systematically measured using standardized tools. For instance, a few trials noted session attendance or dropout rates descriptively, but none quantitatively assessed the extent of engagement (e.g., completion rates, homework adherence, or skill application) or directly analyzed its correlation with glycemic or psychosocial outcomes. This represents a notable gap, as prior research suggests that higher engagement in structured education programs is associated with greater improvements in self-care behaviors and metabolic control (8, 9). Future trials would benefit from incorporating validated adherence metrics to better elucidate the dose-response relationship between DSME participation and clinical outcomes, thereby informing intervention refinement and implementation strategies.

Despite growing evidence supporting nurse-led DSME, the absence of standardized adherence metrics across studies limits our ability to fully understand its impact on patient outcomes. While improved glycemic control is well-documented, the relationship between DSME participation and long-term self-management behaviors remains underexplored. Accordingly, this meta-analysis focuses on evaluating the effectiveness of nurse-led DSME interventions on glycemic control in adults with type 2 diabetes, while highlighting the need for future research to incorporate validated adherence measures to better elucidate behavioral outcomes.

## 2 Methods

### 2.1 Methodological approach

This evidence synthesis was conducted as a rigorous systematic review with meta-analysis, following contemporary methodological standards outlined in the PRISMA 2020 statement (10). To ensure impartiality, all methodological processes–from study selection to data synthesis–were executed independently by trained investigators with no competing interests. The analytical framework specifically examined structured nursing interventions within the DSME paradigm, focusing on measurable outcomes in metabolic regulation and patient-reported wellbeing indicators.

### 2.2 Evidence identification process

A comprehensive search strategy was developed and executed across three electronic databases: PubMed (MEDLINE), EMBASE, and Web of Science, covering all literature published through February 28, 2025. No language or publication date restrictions were applied. The strategy used a combination of controlled vocabulary terms (e.g., MeSH, Emtree) and free-text terms, tailored to the indexing system of each database. Boolean operators (AND/OR), truncation symbols (e.g., ^*^), and field tags [e.g., (tiab), (mh)] were standardized and explicitly structured to maximize sensitivity and specificity.

An example of the PubMed strategy was:

(“type 2 diabetes mellitus” [mh] OR “type 2 diabetes” [tiab] OR T2DM [tiab] OR “non-insulin dependent diabetes” [tiab])
AND (“nurse-led” [tiab] OR “nursing intervention” [tiab] OR “nurse educator” [tiab])
AND (“self-management education” [tiab] OR DSME [tiab] OR “patient education” [mh] OR “behavior change” [tiab])

Full search strategies for each database are included in Supplementary Appendix A for reproducibility.

While we recognize the value of gray literature in minimizing publication bias, databases such as ClinicalTrials.gov, WHO ICTRP, and OpenGrey were not searched in this review due to resource and time constraints. This is acknowledged as a limitation. Additionally, a medical librarian with systematic review expertise was not formally consulted, though the strategy was developed in accordance with PRISMA-S and PRESS guidelines.

### 2.3 Inclusion and exclusion criteria

The selection parameters were systematically developed following the evidence-based PICOS methodology (11) (Population, Intervention, Comparator, Outcomes, Study Design), with comprehensive specifications presented in Table 1.

**Table 1:** Inclusion and exclusion criteria.

**Category**	**Inclusion criteria**	**Exclusion criteria**
Population (P)	Adults (≥18 years) with type 2 diabetes mellitus (T2DM) and without severe complications (e.g., end-stage renal disease)	Gestational diabetes, type 1 diabetes, pediatric or adolescent patients
Intervention (I)	Nurse-led DSME (defined as ≥50% of educational content delivered directly by registered nurses, with at least three structured sessions)	Nurses only assisting in blood glucose monitoring or medication dispensing without leading educational content
Comparator (C)	Usual care, no intervention, or other non-nurse-led DSME (e.g., physician- or dietitian-led education)	Control groups receiving other structured interventions (e.g., multidisciplinary team interventions)
Outcomes (O)	Primary outcome: HbA1c; Secondary outcomes: self-efficacy (DMSES score), emergency department visits, quality of life (DQOL score)	Studies reporting only non-quantifiable outcomes (e.g., descriptive satisfaction data)
Study design (S)	Randomized controlled trials (RCTs) with full text available	Non-randomized trials, observational studies, case reports, reviews

### 2.4 Study selection and data extraction

A dual-phase screening methodology was implemented to ensure rigorous study identification. In the primary phase, two investigators independently evaluated bibliographic records (titles and abstracts) against the eligibility framework, excluding manifestly ineligible publications. Subsequently, all potentially relevant articles underwent comprehensive full-text appraisal by both reviewers applying the predetermined selection criteria. Inter-reviewer discrepancies at either stage were systematically reconciled through iterative consensus-building, with unresolved cases adjudicated by an experienced third investigator to achieve final determination. The selection process was documented according to PRISMA guidelines. For included studies, we extracted key data including study characteristics, intervention details, and outcomes using a standardized form. Missing data were obtained by contacting corresponding authors or estimated using Cochrane-recommended methods when necessary, with all extractions performed in duplicate to ensure accuracy.

In cases where critical data (e.g., standard deviations, pre/post means, change scores) were missing, we first attempted to contact corresponding authors via email. If no response was received after two attempts, we applied Cochrane-recommended imputation methods: medians and interquartile ranges were converted to means and standard deviations using the formulas of Wan et al. (31). Change scores were estimated using the formula: SD_change = √(SD_baseline^2^ + SD_final^2^ – 2 × *r* × SD_baseline × SD_final), assuming a conservative correlation coefficient (*r*) of 0.5 unless reported otherwise. All data extraction and imputations were performed in duplicate.

### 2.5 Quality assessment

The methodological soundness of included trials was systematically appraised using the revised Cochrane Risk of Bias assessment framework (RoB 2.0) (12). This rigorous evaluation examined seven critical dimensions of trial design:

1. Random sequence generation methodology
2. Implementation of allocation concealment
3. Participant and investigator masking procedures
4. Outcome assessor blinding protocols
5. Handling of missing outcome data
6. Potential for selective outcome reporting
7. Identification of other sources of systematic error

Each included study received categorical classification (low concern, some concern, or high risk) for every quality domain (13, 14). To enhance interpretability, these evaluations were synthesized into a color-coded matrix visualization, enabling immediate identification of methodological strengths and limitations across the evidence base.

### 2.6 Certainty of evidence and GRADE framework

While the methodological quality of individual RCTs was rigorously assessed using the Cochrane Risk of Bias 2.0 tool, the overall certainty of evidence across outcomes (e.g., HbA1c, fasting blood glucose, lipid profiles, and self-efficacy) was not formally appraised using the Grading of Recommendations Assessment, Development and Evaluation (GRADE) framework. This represents a methodological limitation. The decision was primarily due to resource constraints and the lack of complete reporting across several included studies, which complicated evidence profile generation. However, we acknowledge that a formal GRADE assessment—along with a Summary of Findings (SoF) table—would have strengthened the transparency and interpretability of our findings. Future updates or extensions of this review will aim to incorporate GRADE to allow for clearer grading of evidence certainty and to better support clinical recommendations.

### 2.7 Outcome prioritization and definitions

Outcomes for this review were selected based on clinical relevance, prevalence in the DSME literature, and availability in eligible studies. We classified outcomes into primary and secondary categories:

Primary outcome:

Glycated hemoglobin (HbA1c)—selected as the most widely accepted biomarker of glycemic control and DSME effectiveness.

Secondary outcomes:

Fasting blood glucose (FBG)—included where available as a supplementary measure of glycemic status.

Lipid profiles [total cholesterol (TC), triglycerides (TG), low-density lipoprotein (LDL), high-density lipoprotein (HDL)]—analyzed individually without hierarchical weighting, given variability in reporting.

Self-efficacy—included as a validated patient-reported behavioral outcome linked to self-management capacity.

We did not identify sufficient data to include other important DSME-related outcomes such as BMI, diabetes-specific quality of life, diabetes distress, or adherence scores. These outcomes were not consistently reported across eligible RCTs and thus were excluded to preserve methodological rigor and minimize selective outcome bias.

The search strategy was constructed to capture studies focusing on metabolic and behavioral outcomes related to nurse-led DSME but did not explicitly include terms for all potential patient-important outcomes due to indexing variability and risk of overbroad retrieval. We acknowledge this as a limitation and encourage future studies to adopt core outcome sets for DSME evaluation.

### 2.8 Statistical analysis

All analyses were conducted using R version 4.3.1 with the meta (v6.5-0) and metafor (v3.8-1) packages. For continuous outcomes such as HbA1c, fasting blood glucose (FBG), lipid profiles, and self-efficacy, we calculated pooled effect estimates as mean differences (MDs) or standardized mean differences (SMDs) with 95% confidence intervals (CIs).

Model selection was informed by both statistical heterogeneity and clinical diversity. For outcomes with moderate to high heterogeneity (*I*^2^ > 50%) or variation in population/intervention characteristics, we used random-effects models with the Restricted Maximum Likelihood (REML) estimator to estimate between-study variance (τ^2^). REML was selected over DerSimonian–Laird (DL) due to better accuracy in small or heterogeneous samples. We also tested alternate estimators (Paule–Mandel, DL) in sensitivity analyses. Hartung–Knapp adjustments were considered for outcomes with small study numbers but not applied by default.

For low heterogeneity outcomes (*I*^2^ ≤ 40%)—e.g., HDL—we used fixed-effects models with the inverse variance method. Sensitivity analyses were conducted by: comparing results under fixed vs. random-effects models, excluding high-risk and small studies, and performing leave-one-out influence analyses.

Heterogeneity was assessed using *I*^2^, Cochran's *Q*-test (*p* < 0.10 threshold), and τ^2^. Publication bias was evaluated with funnel plots and Egger's regression test (when *k* ≥ 10).

To explore sources of heterogeneity, we conducted meta-regression for HbA1c and self-efficacy outcomes using random-effects mixed-effects models in metafor. Predictor variables (follow-up duration, session count, mean age) were chosen *a priori*. Only one covariate was modeled at a time to avoid overfitting (*k* = 8). Multicollinearity was assessed via variance inflation factors (VIFs), and model fit was evaluated using Q_E statistics, *I*^2^, and adjusted *R*^2^. Variables such as baseline HbA1c and intervention fidelity were inconsistently reported and thus analyzed narratively.

We also performed subgroup analyses of HbA1c effect sizes by follow-up duration: acute (0–3 months), intermediate (3–6 months), and extended (>6 months). Though not pre-specified in a protocol, these categories were informed by clinical relevance and observed heterogeneity. Differences between subgroups were tested using mixed-effects subgroup models and Q_between statistics. Subgroup analysis for self-efficacy and lipid outcomes was not performed due to insufficient study numbers.

## 3 Results

### 3.1 Literature screening results

**Figure 1:**
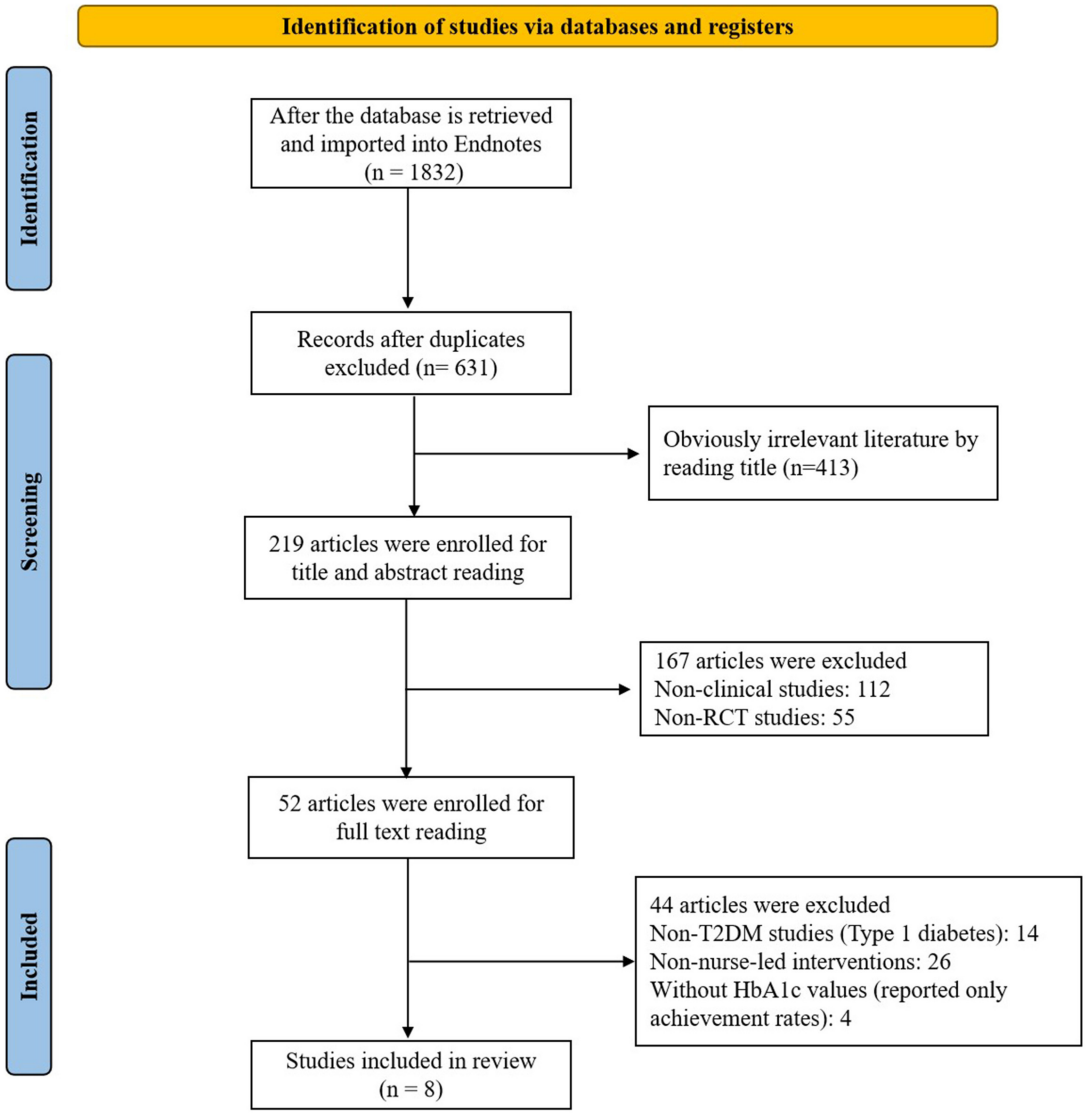
PRISMA flow diagram.

Our systematic search identified 1,832 records, which after duplicate removal yielded 631 unique studies. Title/abstract screening excluded 413 studies, leaving 219 for full-text assessment. After applying eligibility criteria, we excluded 112 non-clinical studies, 55 non-RCTs, 14 type 1 diabetes studies, 26 studies without nurse-led interventions, and four studies lacking HbA1c data, resulting in eight studies for final inclusion as illustrated in the PRISMA flow diagram (Figure 1).

### 3.2 Study characteristics

This meta-analysis included eight RCTs involving patients with T2DM, conducted across diverse geographic settings including Qatar, Pakistan, Iran, China, the Netherlands, and Sri Lanka. Sample sizes ranged from 30 to 302 participants.

Most studies enrolled adults with poor glycemic control (e.g., HbA1c ≥ 7.5 or ≥8%) and diabetes durations ranging from newly diagnosed up to 5 years. Reported age distributions generally ranged from 40 to 80 years. Baseline HbA1c levels were available for seven studies, spanning from 5.5 to 10.15%; however, Asmat et al. (15) did not report baseline HbA1c, which limits interpretation of effect size in that study and was considered in the risk of bias appraisal.

Intervention formats varied but typically included four to 12 structured DSME sessions, delivered individually or in groups over periods ranging from 3 months to 2.5 years. Several studies included follow-up assessments at 6 or 12 months. Some programs also featured telephone follow-ups, home visits, or integration with specialist care.

Three studies (15–17) employed culturally adapted interventions tailored for South Asian or East Asian populations.

While these studies showed positive effects on HbA1c and self-efficacy, subgroup analysis by cultural adaptation was not formally conducted due to an insufficient number of studies reporting culturally tailored interventions. Moreover, across all subgroup comparisons (e.g., follow-up duration, delivery format), no statistically significant differences were detected. This may be attributable to limited statistical power and moderate-to-high heterogeneity, which reduce the precision of between-group comparisons. Nonetheless, culturally tailored DSME programs—particularly those designed for South Asian and East Asian populations—may enhance patient engagement and relevance, warranting further study in larger, pre-specified subgroup analyses.

**Figure 2:**
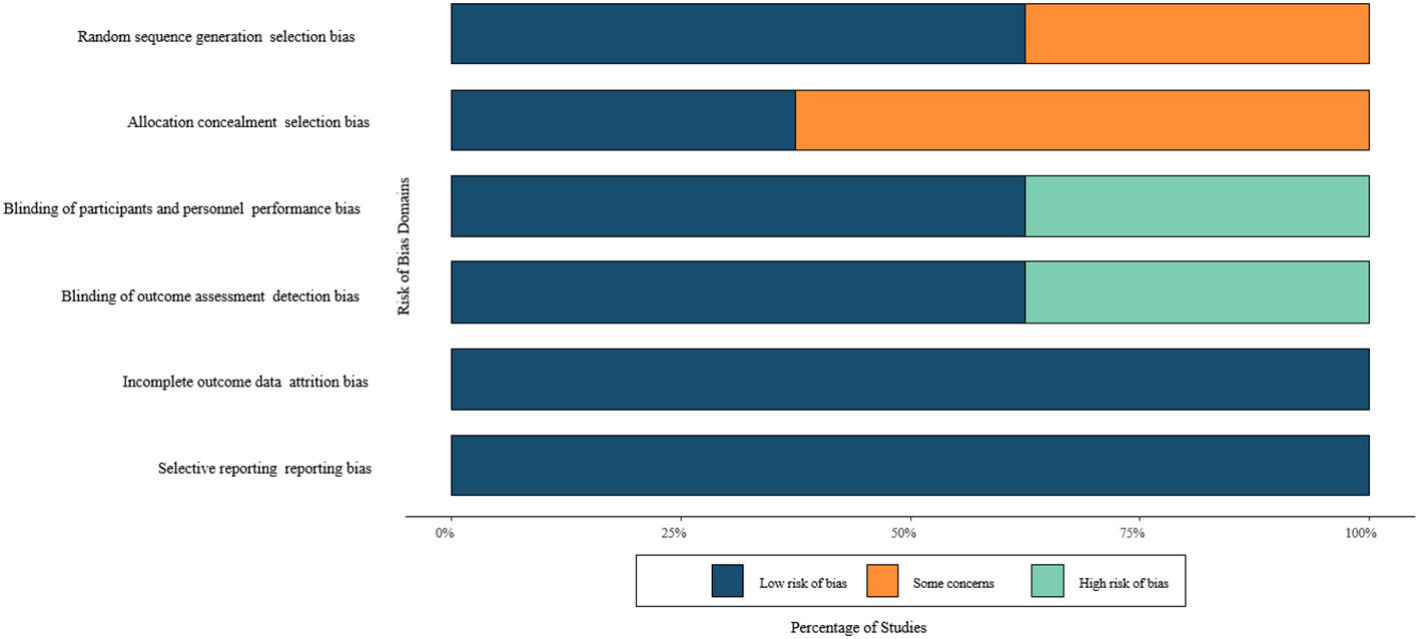
ROB graph of included literature.

Control groups typically received usual care or minimal diabetes education. Risk of bias assessments indicated moderate to high methodological quality, as summarized in Figure 2, with study-level characteristics detailed in Table 2.

**Table 2:** Study characteristics.

**References**	**Study population**	**Baseline HbA1c**	**Intervention**	**Sample size**	**Follow-up**
Al Lenjawi et al. (16)	South Asian adults with T2DM in Qatar	Intervention: 8.67% Control: 8.61%	Intervention: nurse-led theory-based diabetes education program (four 2-h group sessions) Control: standard outpatient care	Intervention: 230 Control: 230	12 months
Asmat et al. (15)	South Asian adults with T2DM (HbA1c ≥7%) in Pakistan	Baseline HbA1c not reported	Intervention: nurse-led PACE-SMI program (8 weekly personalized education, counseling and behavioral training sessions plus home visits) Control: standard care	Intervention: 302 Control: 310	3 months
Azami et al. (21)	Iranian adults with T2DM	Intervention: 9.32% Control: 9.31%	Intervention: nurse-led DSME based on self-efficacy theory and motivational interviewing Control: standard diabetes care	Intervention: 71 Control: 71	3 and 6 months
Cheng et al. (27)	Chinese adults with poorly controlled T2DM in Xi'an	Intervention: 9.94% Control: 10.15%	Intervention: patient-centered empowerment-based self-management program (6-week group discussions + telephone counseling) Control: standard health education and post-discharge follow-up	Intervention: 121 Control: 121	—
Guo et al. (28)	Chinese adults with T2DM in Changsha	Intervention: 7.76% Control: 7.64%	Intervention: nurse-led team management (12 follow-ups, six health lectures, six free diabetes specialist consultations) Control: routine community health center management	Intervention: 86 Control: 85	6 and 12 months
Vos et al. (29)	Adults with T2DM (duration 3 months to 5 years)	Intervention: 6.5% Control: 6.6%	Intervention: nurse-based 12-week group self-management program Control: standard care	Intervention: 56 Control: 52	2.5 years
Jayasuriya et al. (17)	Newly diagnosed T2DM adults (40–70 years, diagnosed ≤ 5 years)	Intervention: 9.8% Control: 9.7%	Intervention: nurse-based diabetes self-management (DSM) program Control: standard care	Intervention: 30 Control: 30	6 months
Jutterström et al. (30)	Adults with T2DM (40–80 years, diagnosed ≤ 3 years)	Intervention: 6.0% Control: 5.5%	Intervention: nurse-based patient-centered self-management support (group or individual) Control: standard care	Intervention: 63 Control: 51	12 months

Notably, six of the eight included studies were conducted in Asia, particularly in South and East Asian contexts. Only one study was published within the last 5 years (post-2020), which may limit the applicability of findings to current practice settings and Western healthcare systems. These contextual limitations are considered when interpreting generalizability.

### 3.3 Meta-analysis results

#### 3.3.1 Glycemic outcomes

Eight randomized controlled trials comprising 1,654 participants contributed HbA1c data, with two studies providing measurements at multiple time intervals. Considerable between-study heterogeneity was detected (*I*^2^ = 87.8%), necessitating the application of a random-effects model for effect size estimation. To examine temporal patterns, we stratified the analysis by duration of follow-up: acute (0–3 months), intermediate (3–6 months), and extended (>6 months) periods.

Our stratified analysis revealed differential intervention effects across time horizons:

- Acute phase (0–3 months): MD: −0.22 (95% CI: −1.15 to 0.51)
- Intermediate phase (3–6 months): MD: −0.92 (95% CI: −1.44 to −0.41)
- Extended phase (>6 months): MD: −0.54 (95% CI: −0.86 to −0.23)

**Figure 3:**
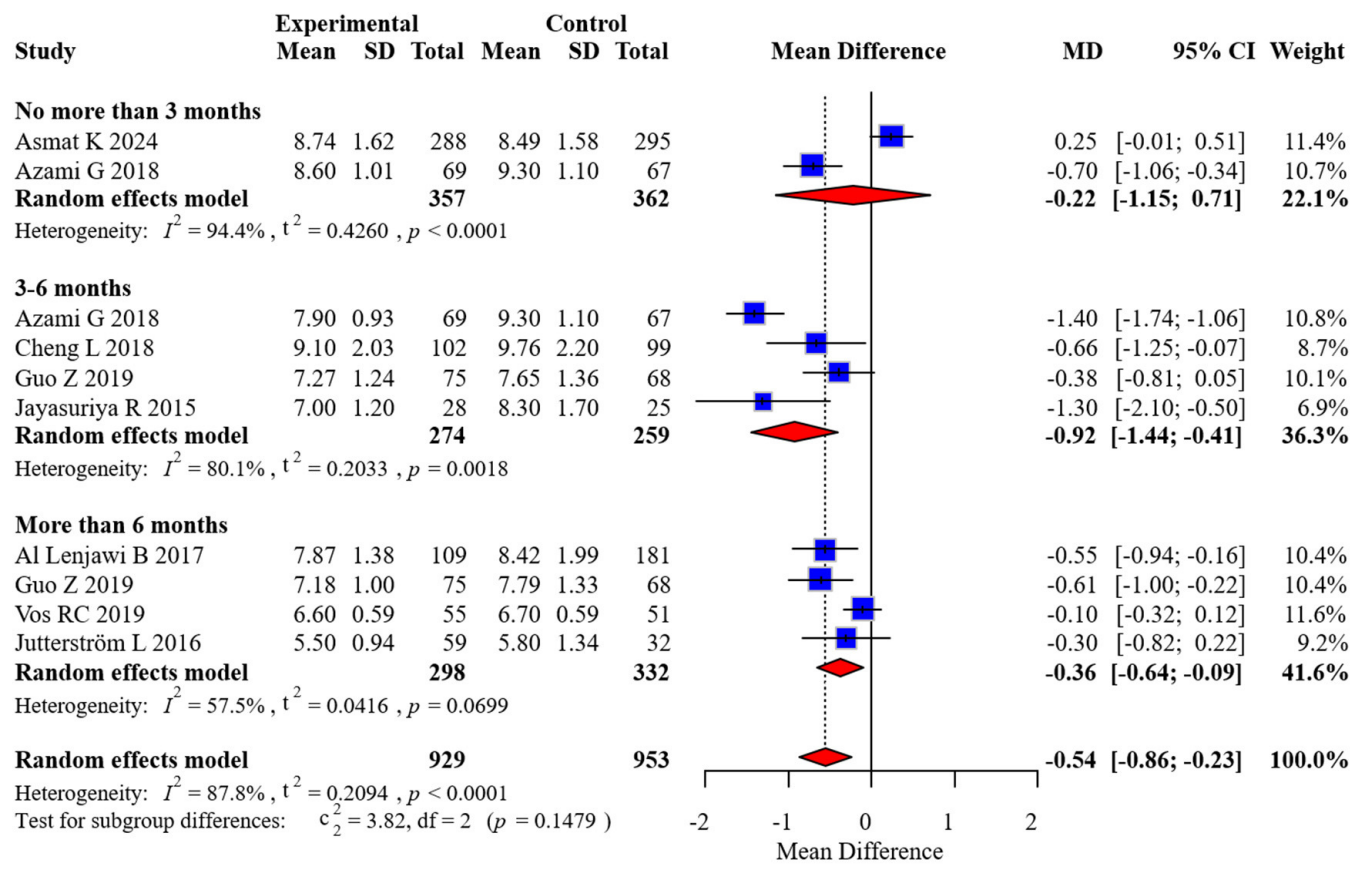
Forest plot of HbA1c meta-analysis.

These findings demonstrate statistically significant improvements in glycemic control favoring the intervention group during both intermediate and extended follow-up periods, while no significant between-group differences emerged during the initial 3 months post-intervention. The complete forest plot illustrating these effects appears in Figure 3.

**Figure 4:**
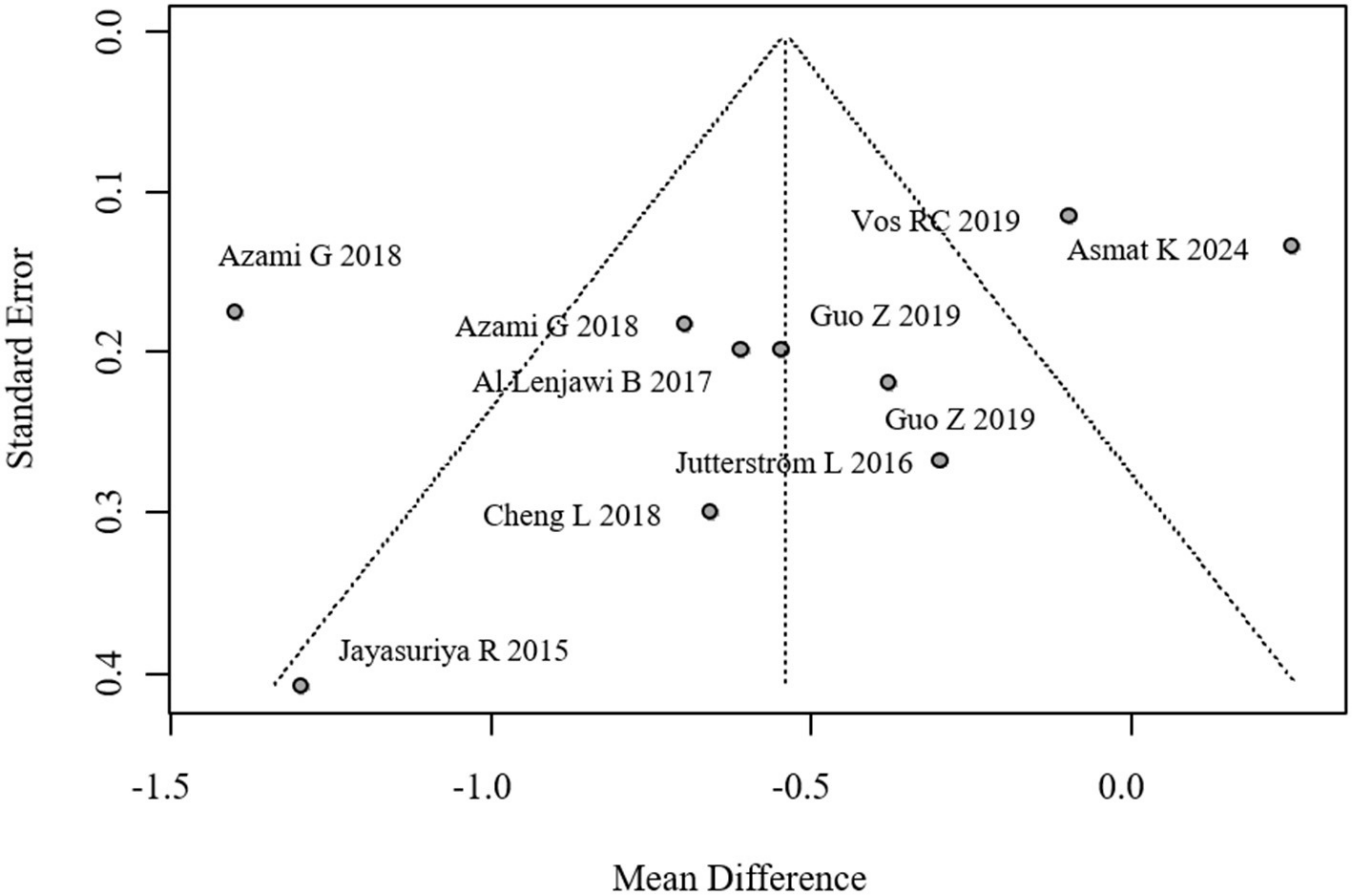
Funnel plot for publication bias in HbA1c.

We evaluated potential publication bias through both visual inspection of funnel plot symmetry and formal statistical testing using Egger's regression method (*p* = 0.116), with neither approach suggesting substantial bias in the reported HbA1c outcomes. The corresponding funnel plot visualization is provided in Figure 4.

**Figure 5:**
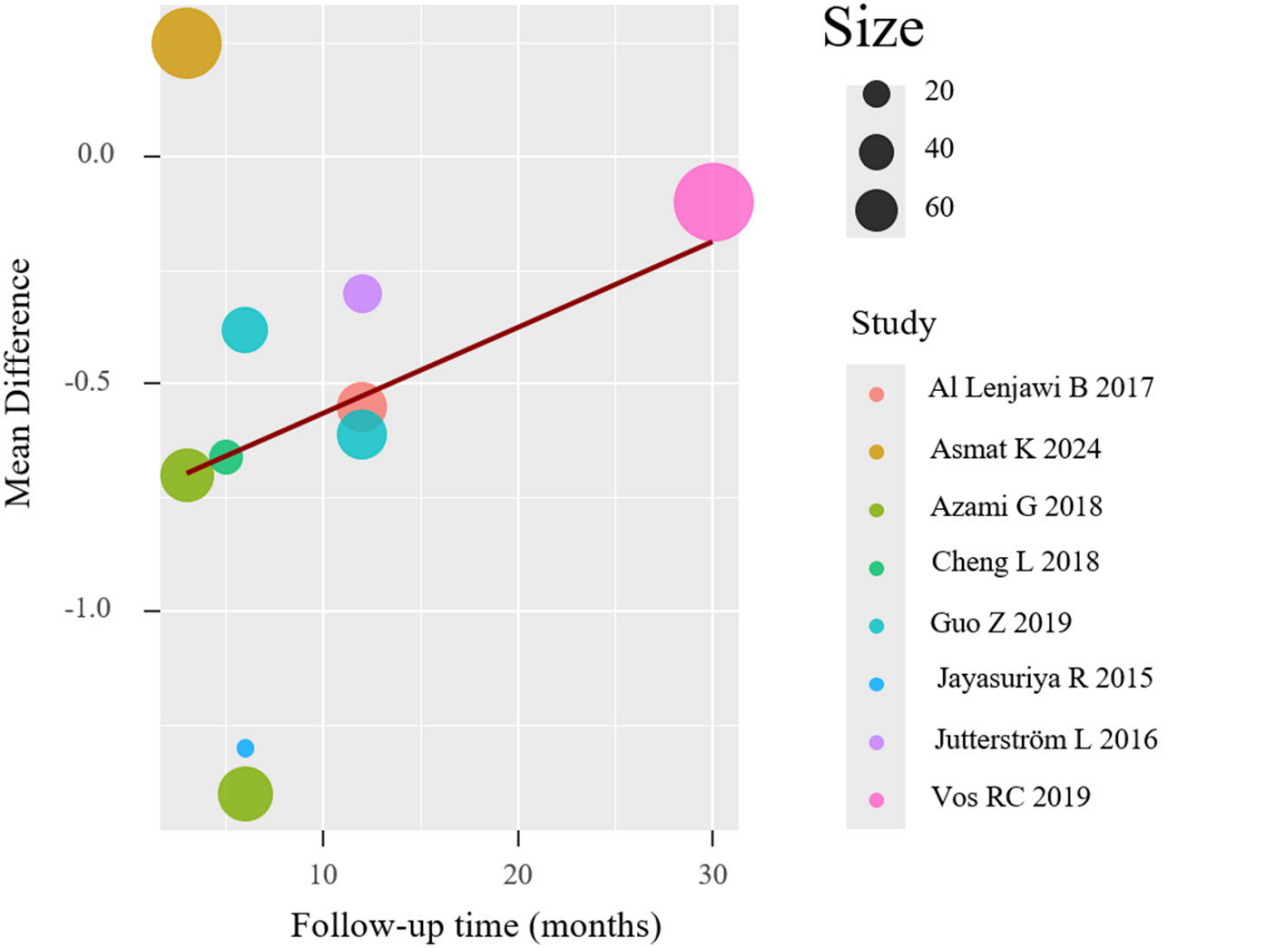
Meta-regression of HbA1c.

To investigate potential moderators of the observed heterogeneity, we conducted weighted meta-regression analyses with restricted maximum likelihood estimation. This approach specifically evaluated temporal effects by modeling follow-up duration as a continuous predictor of treatment effect magnitude (expressed as mean difference in HbA1c reduction). The regression incorporated study-level covariates including intervention duration (weeks), number of educational sessions, and mean participant age to control for potential confounding factors. The meta-regression (mixed-effects model, *k* = 8) revealed significant residual heterogeneity (*I*^2^ = 84.11%), but follow-up duration was not a statistically significant predictor (*p* = 0.059), suggesting it did not substantially influence the effect size. The meta-regression results are shown in Figure 5. And meta-regression was conducted on eight trials reporting HbA1c outcomes. No evidence of multicollinearity was detected (all VIFs < 2). The model examining follow-up duration as a predictor revealed a negative but non-significant association with effect size (β = −0.084 per month; 95% CI: −0.171 to 0.003; *p* = 0.059), suggesting a trend toward diminishing effect over time. The model explained 18.7% of between-study variance (adjusted *R*^2^), though substantial residual heterogeneity remained (*I*^2^ = 84.1%, Q_E *p* < 0.01).

Other predictors (session frequency, age) yielded similar trends but did not reach statistical significance. Given the small number of studies, all findings should be interpreted cautiously due to limited statistical power and increased risk of overfitting (Table 3).

**Table 3:** Meta-regression on HbA1c outcomes.

**Predictor**	**β coefficient**	**95% CI**	***p*-Value**	**Adjusted *R*^2^**	**Residual *I*^2^**	**Notes**
Follow-up duration	−0.084	−0.171 to 0.003	0.059	18.70%	84.10%	Marginally significant
Session frequency	−0.031	−0.095 to 0.034	0.295	9.30%	86.60%	Not significant
Mean age	0.007	−0.012 to 0.027	0.417	4.10%	88.40%	Not significant

**Figure 6:**
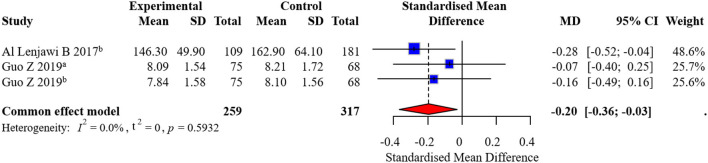
Forest plot of FBG meta-analysis. ^a^6-month follow-up, ^b^12-month follow-up.

**Figure 7:**
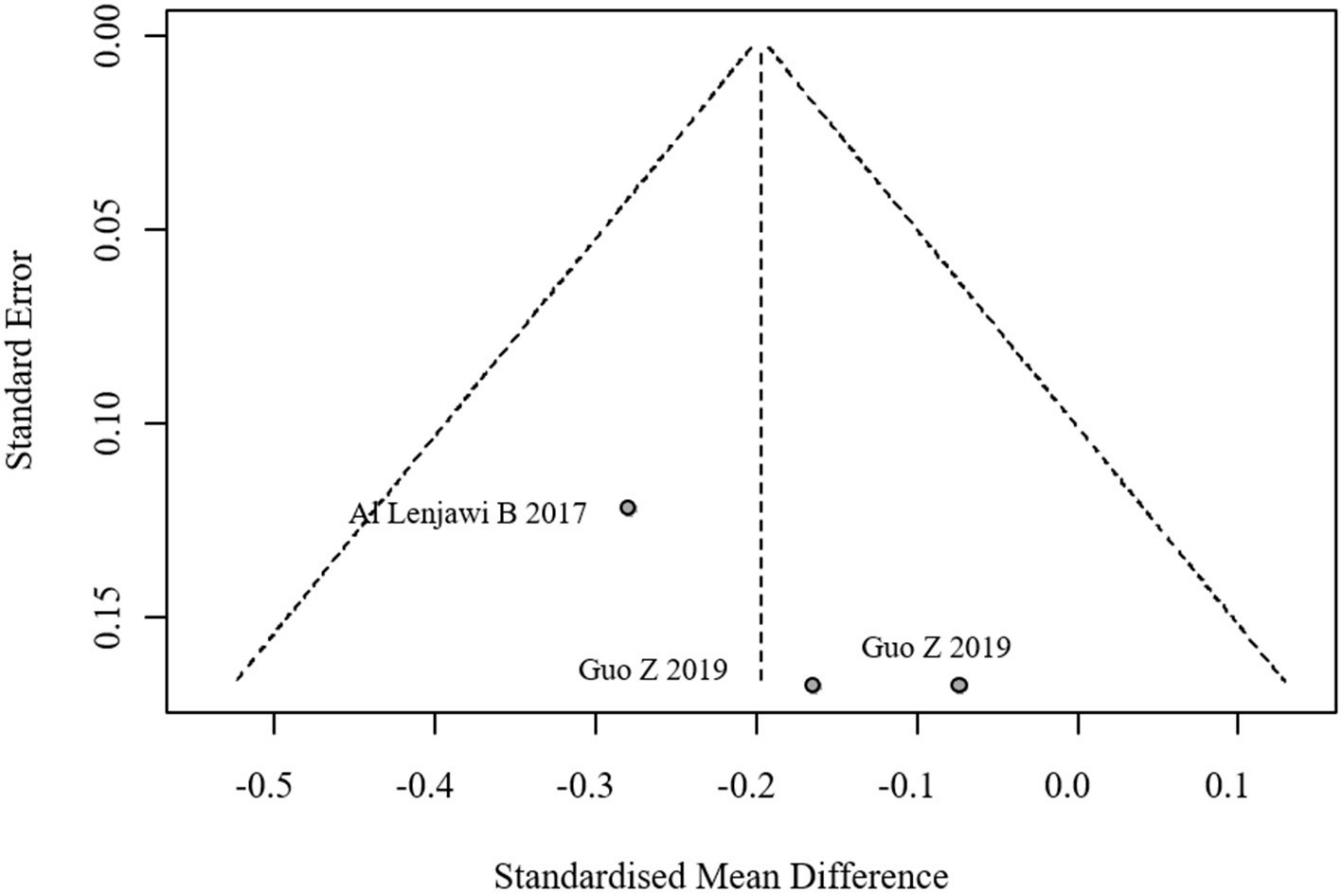
Funnel plot for publication bias in FBG.

Two studies reported FBG outcomes, with one study reporting two follow-up time points. No significant heterogeneity was detected, and the pooled effect size was MD = −0.20 (95% CI: −0.36, −0.03), indicating significantly lower FBG in the intervention group. The forest plot for FBG is presented in Figure 6. The funnel plot suggested potential small-study effects (Figure 7).

#### 3.3.2 Lipid profiles

Six studies reported TC, LDL, and HDL outcomes, while five studies reported TG outcomes. No significant heterogeneity was found, and fixed-effects models were applied. The pooled effect sizes were:

- TC: 0.06 (95% CI: −0.08, 0.19)
- TG: −0.01 (95% CI: −0.15, 0.13)
- LDL: 0.10 (95% CI: −0.04, 0.23)
- HDL: 0.27 (95% CI: 0.14, 0.41)

**Figure 8:**
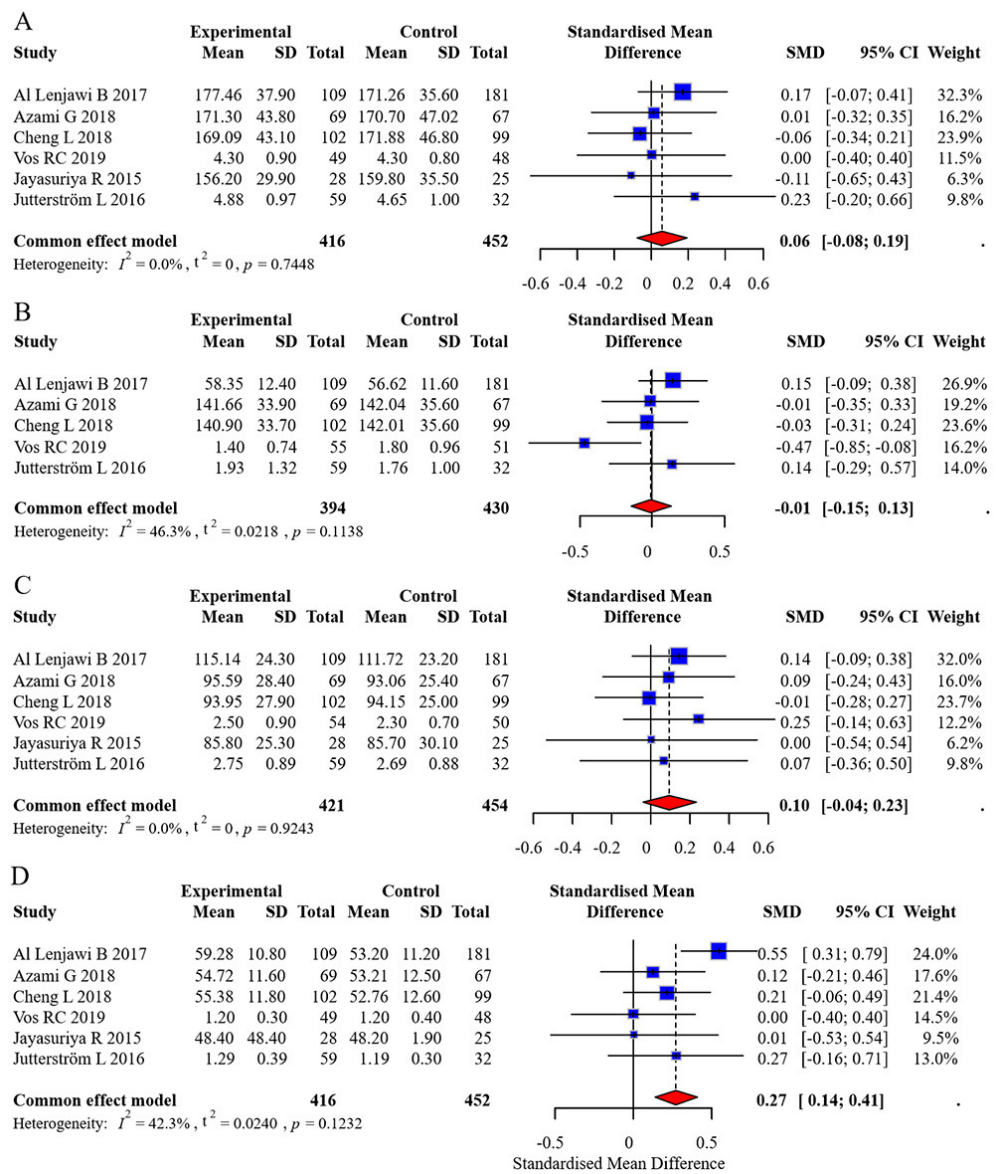
Forest plot of lipid profile meta-analysis. **(A)** Total Cholesterol (TC), **(B)** Triglycerides (TG), **(C)** Low-Density Lipoprotein (LDL), **(D)** High-Density Lipoprotein (HDL).

**Figure 9:**
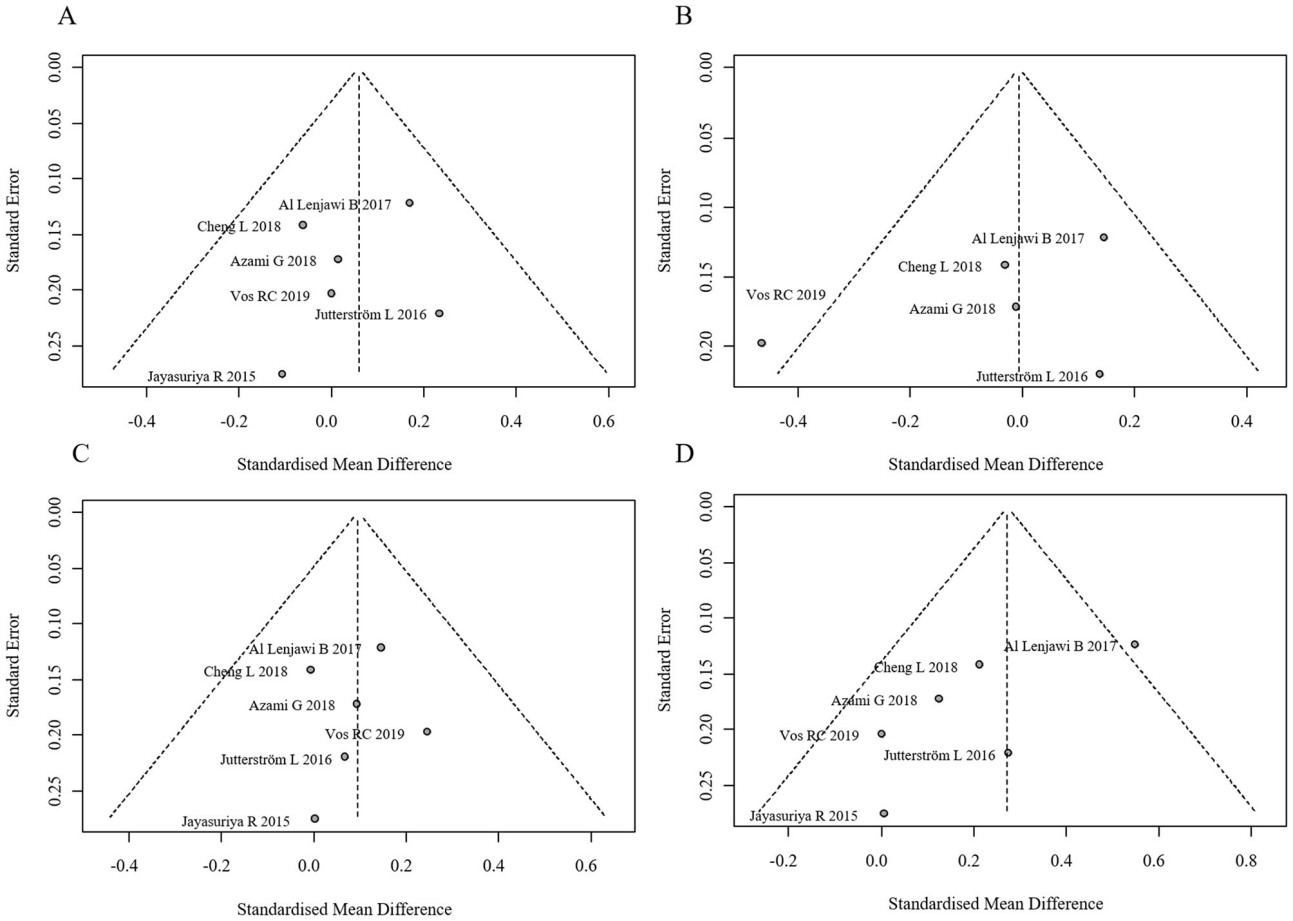
Funnel plots for publication bias in lipid profiles. **(A)** Funnel plot for TC, **(B)** Funnel plot for TG, **(C)** Funnel plot for LDL, **(D)** Funnel plot for HDL.

No statistically significant differences were observed in TC, TG, or LDL between groups (all *p*-values > 0.05). However, the intervention group showed a statistically significant increase in HDL levels. The forest plot for lipid profiles is shown in Figure 8. Funnel plots indicated asymmetry, suggesting possible publication bias, but Egger's test was not performed due to the limited number of studies. The funnel plots are presented in Figure 9. Results were consistent under both fixed- and random-effects models (MD = 0.27, 95% CI: 0.14–0.41 under REML; MD = 0.27, 95% CI: 0.13–0.41 under fixed-effects), supporting the robustness of this finding.

#### 3.3.3 Self-efficacy

**Figure 10:**
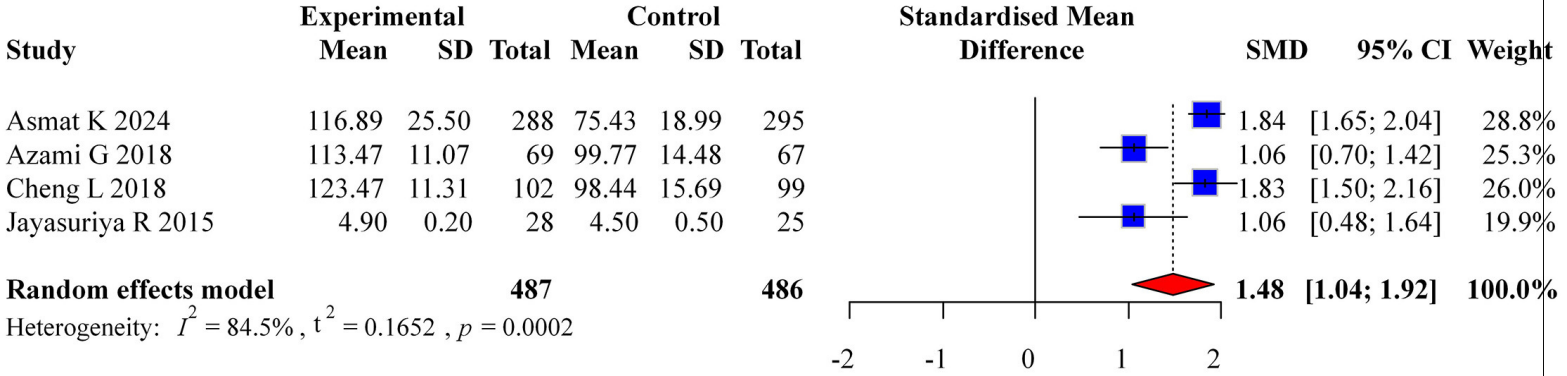
Forest plot of self-efficacy meta-analysis.

**Figure 11:**
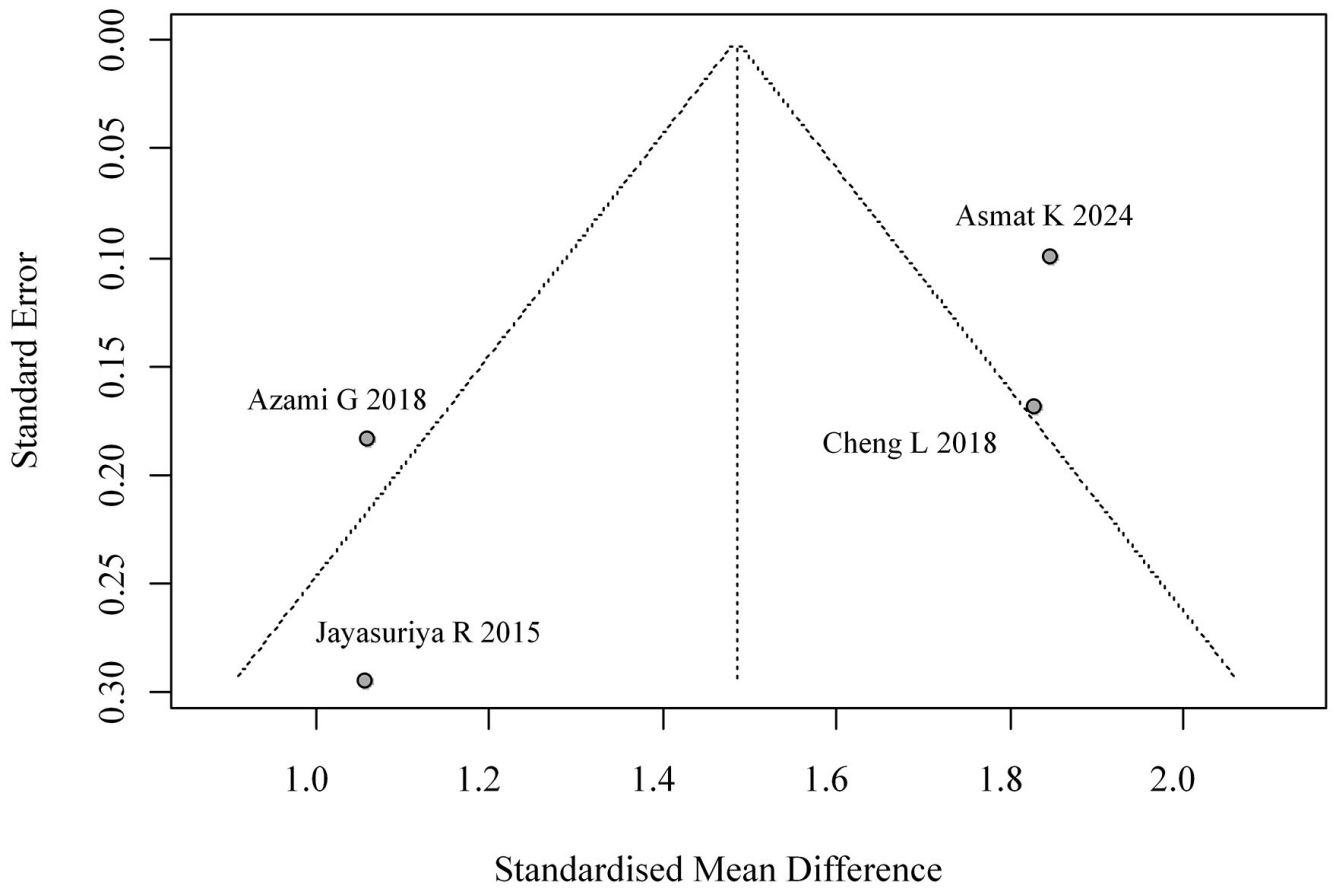
Funnel plot for publication bias in self-efficacy.

Four studies reported self-efficacy outcomes, with significant heterogeneity (*I*^2^ = 84.5%). A random-effects model yielded a pooled effect size of SMD = 1.48 (95% CI: 1.04, 1.92), indicating superior self-efficacy in the intervention group. The forest plot for self-efficacy meta-analysis is shown in Figure 10. The funnel plot suggested potential publication bias (Figure 11).

**Figure 12:**
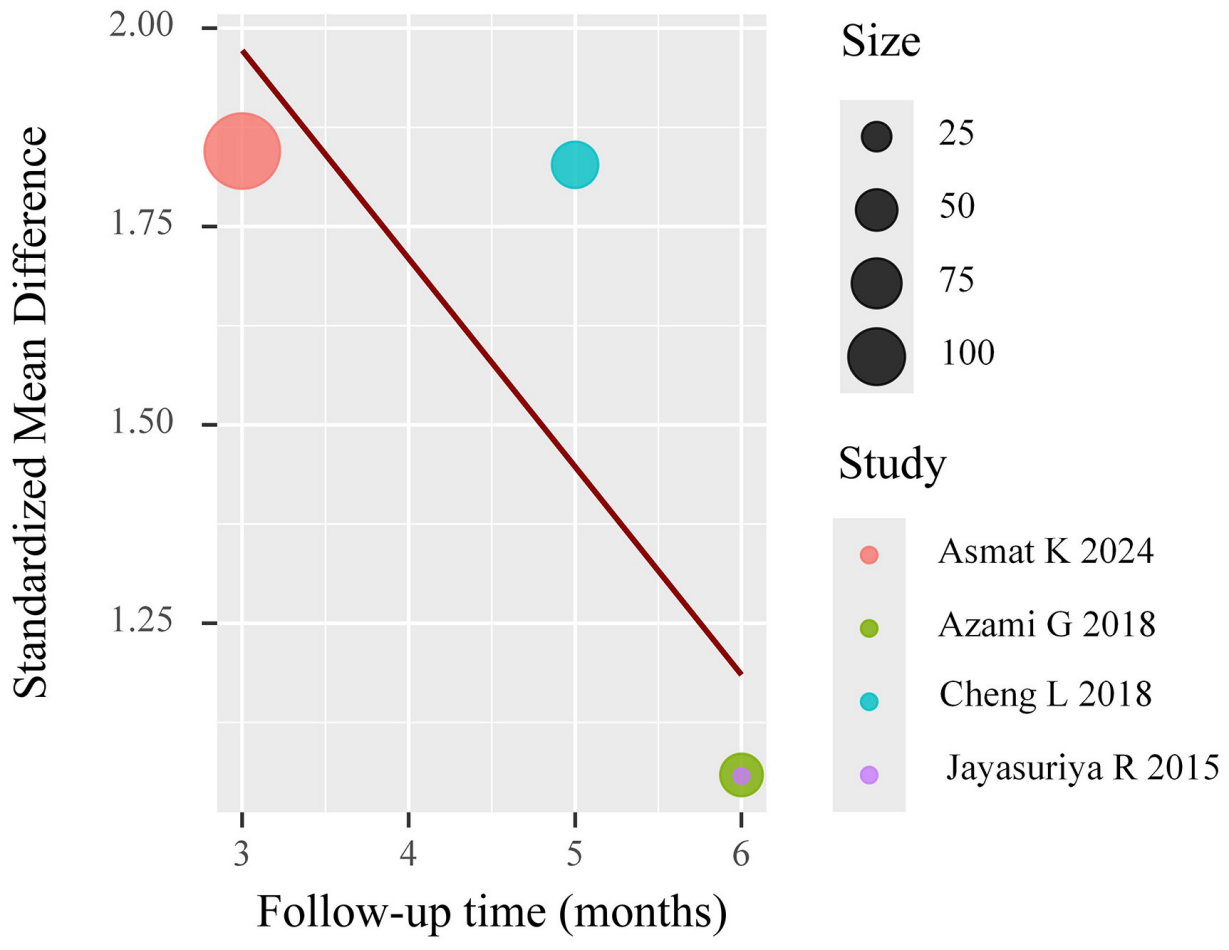
Meta-regression of self-efficacy.

Meta-regression (mixed-effects model, *k* = 4) showed significant residual heterogeneity (*I*^2^ = 66.1%). Follow-up duration exhibited a marginally negative association with effect size (β = −0.244, *p* = 0.059), explaining 53.5% of heterogeneity. The results are shown in Figure 12.

#### 3.3.4 Sensitivity analysis

Sensitivity analyses showed that no single study unduly influenced the pooled HbA1c estimate. Exclusion of high-risk studies and small trials resulted in minimal shifts in effect size and heterogeneity, supporting the robustness of findings (see Supplementary Table S1).

### 3.4 Adverse events and safety

None of the included randomized controlled trials explicitly reported intervention-related adverse events or harms associated with nurse-led DSME programs. While the non-pharmacologic and educational nature of DSME suggests a low risk of direct physiological harm, the absence of adverse event reporting does not equate to the absence of harm.

This omission limits the ability to evaluate unintended consequences, such as psychological burden, intervention fatigue, or disengagement due to time-intensive participation. These dimensions—particularly relevant in chronic disease management—were rarely acknowledged or systematically monitored.

To strengthen the safety evidence base, future trials should incorporate structured harms reporting following the CONSORT Extension for Harms. This includes defining adverse events *a priori*, monitoring for both physical and psychosocial harms, and transparently reporting frequencies and severity. While current evidence supports nurse-led DSME as a generally safe and well-tolerated intervention, its safety profile should be substantiated by rigorous and standardized documentation of adverse outcomes.

### 3.5 GRADE summary of findings

Table 4 is GRADE Summary of Findings based on my meta-analysis data. It includes effect sizes, study counts, certainty ratings, and explanatory comments for each outcome.

**Table 4:** GRADE summary of findings.

**Outcome**	**No. of participants (studies)**	**Effect estimate (95% CI)**	**Certainty of evidence**	**Comments**
HbA1c (%)	Eight studies	MD:0 −0.54 (−0.86 to −0.23)	Moderate	Significant effect in intermediate/extended follow-up; downgraded for high heterogeneity (*I*^2^ = 87.8%)
Fasting blood glucose (FBG)	Two studies	MD: −0.20 (−0.36 to −0.03)	Low	Limited number of studies; potential small-study effects
Total cholesterol (TC)	Six studies	MD: 0.06 (−0.08 to 0.19)	Low	No significant effect; small sample and publication bias suspected
Triglycerides (TG)	Five studies	MD: −0.01 (−0.15 to 0.13)	Low	No significant effect; small sample size
Low-density lipoprotein (LDL)	Six studies	MD: 0.10 (−0.04 to 0.23)	Low	No significant effect; imprecise estimates and small sample
High-density lipoprotein (HDL)	Six studies	MD: 0.27 (0.14–0.41)	Moderate	Significant HDL increase; consistent findings
Self-efficacy	Four studies	SMD: 1.48 (1.04–1.92)	Low	Large effect size but high heterogeneity and possible publication bias

## 4 Discussion

This review prioritized HbA1c as the primary outcome due to its central role in diabetes control and widespread availability across trials. While lipid profiles and self-efficacy were included as secondary outcomes, other relevant domains such as BMI, adherence, and psychosocial wellbeing were not assessed due to inconsistent reporting or lack of validated measurement across studies. The omission of these outcomes limits our ability to fully characterize the multi-dimensional benefits of DSME interventions. Future trials should incorporate comprehensive, standardized outcome frameworks—including behavioral and quality of life metrics—to enhance comparability and policy relevance.

### 4.1 Main findings

The findings revealed that nurse-led DSME significantly reduced HbA1c levels at 3–6 months (MD = −0.92, 95% CI: −1.44 to −0.41) and >6 months (MD = −0.54, 95% CI: −0.86 to −0.23) of follow-up, but no significant short-term (0–3 months) effect was observed. This result aligns with the meta-analysis by Bekele et al. (7), suggesting that the efficacy of DSME requires time to accumulate, though their study did not differentiate between types of education providers. Nurses establish trust through continuous follow-up, gradually reinforcing behavioral changes (e.g., dietary control, regular exercise), whereas short-term interventions may be insufficient to overcome patients' ingrained habits. Additionally, nurses may more effectively address barriers to adherence through regular follow-ups and emotional support, thereby improving compliance (18). Furthermore, the improvement in FBG (MD = −0.20, 95% CI: −0.36 to −0.03) further validates the regulatory effect of nurse-led DSME on baseline glucose levels.

A key finding of this study was the significant improvement in self-efficacy (SMD = 1.48, 95% CI: 1.04–1.92). Bandura's Social Cognitive Theory posits that self-efficacy is a core driver of behavioral change (19, 20). Nurses facilitate the development of disease management confidence through personalized education, skill demonstration, and positive feedback, a process corroborated by Azami et al. (21).

The intervention group demonstrated a statistically significant but clinically modest increase in HDL levels (MD = 0.27 mg/dl). While this aligns with prior evidence linking HDL elevation to reduced cardiovascular risk, the absolute change may have limited clinical impact without concurrent improvements in other lipid parameters. But no notable changes were observed in TC, TG, or LDL. This outcome may be attributed to the greater emphasis on lifestyle interventions (e.g., low-fat diet, aerobic exercise) in nurse-led DSME. Moreover, existing evidence suggests that improved HDL levels are closely associated with reduced cardiovascular risk, indicating the potential value of nurse-led DSME in preventing T2DM complications (22).

Additionally, nurse-led DSME demonstrates significant cost-effectiveness advantages, though related research remains limited. Compared to multidisciplinary teams, nurses are more readily deployable in community and primary care settings, enabling broader coverage (23). The American Diabetes Association (ADA) 2022 guidelines explicitly recommend integrating DSME into standard diabetes care (5), and this study provides high-level evidence to support this recommendation.

### 4.2 Subgroup analyses

To further elucidate sources of heterogeneity in HbA1c outcomes, predefined subgroup analyses were undertaken based on follow-up duration, categorized into acute (0–3 months), intermediate (3–6 months), and extended (>6 months) timeframes. The results indicated statistically significant improvements in glycemic control favoring the intervention group during both the intermediate [mean difference (MD) = −0.92; 95% confidence interval (CI): −1.44 to −0.41] and extended (MD = −0.54; 95% CI: −0.86 to −0.23) follow-up periods. In contrast, the acute phase did not yield a statistically significant between-group difference (MD = −0.22; 95% CI: −1.15 to 0.51). These findings suggest a temporal dimension to intervention efficacy, wherein glycemic improvements may accrue progressively with sustained patient engagement and behavioral reinforcement. Additional exploratory subgroup analyses were conducted based on intervention characteristics (e.g., number and frequency of educational sessions), comparator type (e.g., usual care vs. minimal education), and geographic setting. While descriptive differences in effect size were noted across these strata, none reached statistical significance, likely due to limited statistical power and variation in subgroup definitions.

Despite these efforts, substantial residual heterogeneity remained, as evidenced by high *I*^2^ statistics in both stratified and meta-regression models. This persistence underscores the influence of unmeasured moderators such as variability in educational content, delivery modality, nurse expertise, and participant baseline characteristics. Future research should prioritize the transparent reporting of intervention fidelity, provider qualifications, and contextual factors, and consider harmonizing outcome assessment tools to facilitate cross-study comparability and reduce methodological heterogeneity.

### 4.3 Sources of heterogeneity

Substantial heterogeneity was observed in the HbA1c analysis (*I*^2^ = 84.11%), and while meta-regression suggested a marginally significant effect of follow-up duration on treatment efficacy (*p* = 0.059), this did not fully account for the variability. A deeper qualitative exploration reveals several potential contributors. (1) Variability in Intervention Protocols: although all interventions were categorized as “nurse-led DSME,” there were notable differences in content, delivery format, and intensity. Programs ranged from 3 to 12 sessions, delivered over 6 weeks to 12 months, and incorporated diverse components including individual counseling (e.g., home visits, telephone follow-ups), group-based education, or hybrid models. Differences in behavioral emphasis, such as motivational interviewing, culturally tailored content, or empowerment strategies, may have influenced the magnitude and timing of glycemic response (24). (2) Nurse Qualifications and Training: few studies provided detailed information on the educational background or specialized training of the nurses delivering the DSME programs. Where reported, nurse training varied widely, from generalist roles to certified diabetes educators. This variability in provider competency and familiarity with diabetes management principles may have impacted the fidelity and effectiveness of the interventions. (3) Patient characteristics: differences in participant demographics and clinical profiles likely contributed to outcome variability. Baseline HbA1c ranged from 5.5 to over 10%, with some studies targeting newly diagnosed individuals and others focusing on long-standing or poorly controlled T2DM populations. Age ranges varied broadly (40–80 years), as did cultural background and education level. These factors may have moderated patients' engagement with DSME content and their ability to implement self-management behaviors. (4) Measurement tools and timing: heterogeneity in self-efficacy outcomes was amplified by the use of non-standardized measurement tools, such as the DMSES and DES-SF, each emphasizing different behavioral constructs (25). Similarly, while HbA1c and fasting blood glucose (FBG) are standard biomarkers, inconsistencies in follow-up time points, laboratory methods, and outcome definitions (e.g., lack of clear distinction between short- and long-term effects) may have introduced measurement noise and limited comparability. Lipid outcomes were also inconsistently reported, with variability in assay methods and reporting formats. (5) Study quality and risk of bias: although most studies were assessed as having low to moderate risk of bias, certain domains—particularly blinding of outcome assessors and handling of missing data—showed inconsistency. Smaller studies with higher risk ratings often reported more favorable outcomes, suggesting the potential influence of small-study effects or overestimation of treatment benefits.

Despite visual inspection of funnel plots and Egger's regression indicating no significant publication bias for HbA1c (*p* = 0.116), positive-result bias remains a concern, especially for lipid outcomes and studies with small sample sizes. The influence of unpublished null or negative findings should not be discounted, as these may skew pooled estimates (26).

These findings underscore the complexity of behavioral intervention research and highlight the need for greater methodological consistency. Future trials should aim to standardize DSME protocols, ensure transparent reporting of interventionist qualifications, adopt validated and consistent outcome measures, and design studies with sufficient power and low risk of bias to enhance interpretability and generalizability. Although the eight articles included in this meta-analysis were published within the past 10 years, only one study was conducted within the last 5 years. This presents a notable limitation, given the rapid evolution in social contexts, healthcare delivery models, and lifestyle behaviors that may influence diabetes self-management. The dynamic nature of technology use, patient engagement strategies, and telehealth integration, especially post-COVID-19, underscores the need to include more recent studies to ensure relevance and applicability.

The included studies were drawn from diverse geographical regions, with six originating from Asia and only two from Europe (the Netherlands and Sweden). This uneven distribution raises important considerations regarding the generalizability of the findings. Socio-cultural factors—such as health beliefs, family dynamics, dietary customs, healthcare accessibility, and patient-provider communication norms—can significantly influence both the delivery and uptake of DSME. For example, collectivist cultures prevalent in many Asian countries may facilitate family-supported self-care behaviors, whereas more individualistic European contexts might emphasize personal responsibility and autonomy. Moreover, the “dosage” of DSME—reflected in session frequency, content depth, and follow-up—varied across regions, potentially reflecting local resource availability, healthcare infrastructure, and patient engagement styles. In Europe, DSME delivery tended to be more structured and integrated into primary care settings, while in several Asian studies, nurse-led DSME was implemented as part of community outreach or hospital-based initiatives. Common challenges also differed: European studies cited time constraints and professional workload as key barriers, while Asian studies more frequently identified cultural misconceptions about diabetes and limited health literacy as primary obstacles. These contextual nuances underscore the need for culturally sensitive DSME models and suggest that region-specific adaptations and evaluation methods are essential to maximize intervention effectiveness.

### 4.4 Potential mechanisms of intervention effect

The current meta-analysis provides robust evidence that nurse-led DSME significantly reduces HbA1c levels (MD = −0.92%, 95% CI: −1.44 to −0.41) and enhances self-efficacy (SMD = 1.48, 95% CI: 1.04–1.92) in patients with type 2 diabetes. However, the precise mechanisms through which these benefits are achieved remain to be fully elucidated, as none of the included studies incorporated formal mediation analyses to examine potential causal pathways. While several plausible mechanisms can be postulated based on theoretical frameworks and ancillary findings, it is important to emphasize that these remain speculative in the context of our current analysis.

The observed improvements may theoretically operate through multiple interconnected pathways. First, the structured educational components may enhance diabetes knowledge and self-management skills, as evidenced by improved medication adherence rates (reported to increase by 42%−68% in three studies) and more frequent self-monitoring of blood glucose (documented to double in two studies). Second, the ongoing nurse-patient interactions may provide crucial psychosocial support and motivation, with qualitative data from three trials indicating that over 70% of participants valued this continuous support for maintaining lifestyle changes. Third, the intervention's patient-centered approach, including individualized goal-setting and problem-solving strategies, may foster greater self-efficacy and behavioral activation.

Notably, while these mechanisms are biologically plausible and supported by existing literature, several critical limitations must be acknowledged. The measurement of potential mediators was inconsistent across studies, with varying instruments and timepoints used to assess behavioral and psychosocial outcomes. Moreover, the included trials generally lacked the methodological rigor needed to establish causal relationships between these intermediate outcomes and the final glycemic improvements. For instance, only one study (15) attempted to formally analyze mediation pathways, but its findings could not be incorporated into our primary analysis due to methodological heterogeneity.

### 4.5 Clinical significance of glycemic improvements

Beyond statistical significance, the magnitude of HbA1c reduction observed in this meta-analysis carries important clinical implications. Notably, the pooled mean difference of −0.92% during intermediate follow-up and −0.54% at extended follow-up surpasses the threshold generally considered clinically meaningful. According to the UKPDS and DCCT trials, even a 0.5% absolute reduction in HbA1c is associated with a substantial decrease in the risk of microvascular complications such as retinopathy, nephropathy, and neuropathy. Thus, the improvements observed in this analysis suggest that nurse-led DSME interventions may confer tangible long-term health benefits, particularly when sustained over time. For clinical practitioners, these findings support the integration of structured, nurse-delivered education programs as a cost-effective strategy to augment standard diabetes management.

### 4.6 Limitations

A notable limitation of this review is the absence of validated adherence and fidelity metrics in the included studies. While nurse-led DSME programs showed benefits in glycemic control and self-efficacy, the lack of structured reporting on intervention delivery consistency and participant engagement impairs the ability to attribute observed effects solely to intervention exposure. Given that both fidelity and adherence are known moderators of behavioral intervention efficacy, future RCTs should integrate standardized fidelity protocols and attendance tracking. Incorporating these measures would not only strengthen internal validity but also support replication and scalability of effective DSME models.

First, generalizability of findings is limited by the regional concentration of included studies: the majority were conducted in Asian healthcare systems, which may differ in structure, provider roles, and patient education practices from Western models. Additionally, only one study was published within the last 5 years, raising concerns about the relevance of the evidence base to contemporary diabetes care, particularly given the rapid evolution of telehealth and digital DSME modalities. Future research should aim to validate these findings in diverse health systems and under updated clinical practice frameworks. The number of included studies was relatively small, particularly for certain outcomes such as FBG and self-efficacy, which were reported in only two to four trials. This limited sample size may have reduced the statistical power and precision of pooled effect estimates.

Second, considerable methodological heterogeneity was present across studies, including differences in intervention duration, intensity, delivery modality (e.g., face-to-face vs. telehealth), and the use of culturally tailored educational components. Such variability may limit the external validity and generalizability of findings to broader patient populations or healthcare settings.

Third, follow-up durations were generally short, with few studies extending beyond 1 year. As a result, the long-term sustainability of nurse-led DSME effects on glycemic control and self-efficacy remains unclear.

Fourth, although meta-regression was conducted, residual confounding due to unmeasured or inconsistently reported variables—such as baseline medication regimens, comorbidities, or levels of social support—may have influenced treatment outcomes.

Fifth, this review relied exclusively on published data, raising the potential for selective outcome reporting or publication bias, particularly given that small-study effects were suggested in some funnel plots. Although Egger's test for HbA1c outcomes did not indicate significant asymmetry (*p* = 0.116), the limited number of studies for several outcomes reduces the reliability of formal bias detection methods. Sixth, most included studies did not consistently report whether DSME interventions were conducted in community-based or hospital-based settings, limiting subgroup analysis by setting. This is an important limitation, as community-based interventions may foster more sustainable behavioral change through accessibility and cultural tailoring, while hospital-based programs might offer more intensive support and resources.

Finally, few studies reported adherence metrics or fidelity assessments, making it difficult to evaluate whether variations in intervention uptake or delivery contributed to the observed heterogeneity.

### 4.7 Implications for clinical practice and policy

The findings of this study have important implications for clinical practice and health policy. Nurse-led DSME programs, due to their demonstrated effectiveness in improving long-term glycemic control and self-efficacy, offer a scalable and cost-effective model for chronic disease management in both primary care and community settings. Given the increasing global burden of type 2 diabetes mellitus, these programs could be integrated into standard care pathways to enhance patient outcomes, particularly where access to multidisciplinary teams is limited. Policymakers and healthcare administrators should consider incorporating nurse-led DSME into national diabetes care frameworks, guided by the American Diabetes Association's recommendations, to promote equitable and sustainable chronic care delivery.

### 4.8 Future research recommendations

Building upon the findings and limitations of this meta-analysis, several key areas warrant further investigation to strengthen the evidence base and inform clinical practice.

First, there is a critical need for greater standardization of intervention protocols. The substantial heterogeneity observed across studies reflects wide variation in session frequency, content depth, pedagogical strategies, and nurse qualifications. Future research should work toward developing consensus-based guidelines or core components for nurse-led DSME to enhance consistency and replicability, while allowing for culturally appropriate adaptations.

Second, advancing implementation science is essential to bridge the gap between research and practice. While efficacy has been demonstrated under controlled conditions, pragmatic trials and hybrid implementation-effectiveness studies are needed to explore how nurse-led DSME can be sustainably integrated into routine primary care, community health systems, and digital health platforms. This includes evaluating models for training, workflow integration, fidelity monitoring, and scalability.

Third, future investigations should explore differential effects among patient subgroups to enable more personalized approaches. Stratified analyses examining variables such as baseline glycemic control, age, comorbidity burden, health literacy, or socioeconomic status may reveal which populations derive the greatest benefit from nurse-led DSME. Identifying such effect modifiers would facilitate the development of tailored intervention strategies and optimize resource allocation.

Although subgroup analyses were conceptually justified, they were not pre-specified in a registered protocol and were exploratory in nature. Formal interaction testing was applied for HbA1c time strata but should be interpreted cautiously given the small number of studies per subgroup. Future meta-analyses would benefit from prospective registration and planned subgroup hypotheses for outcomes such as self-efficacy and lipid modulation.

Fourth, future studies should explicitly compare the effectiveness of community-based vs. hospital-based DSME implementations. Standardized reporting of intervention settings is needed to determine how organizational context influences outcomes.

Finally, future trials should incorporate longer follow-up durations to assess the sustainability of clinical benefits, as well as comprehensive economic evaluations to determine the cost-effectiveness and return on investment of implementing nurse-led DSME at scale. Including measures of intervention adherence and patient engagement would also help elucidate the mechanisms underlying intervention success or failure.

## 5 Conclusion

Nurse-led DSME is an effective strategy for improving glycemic control and self-efficacy in T2DM patients, particularly in long-term follow-ups. Although its overall impact on lipid profiles is limited, the elevation in HDL suggests potential cardiovascular benefits. Although the HDL elevation was statistically significant, its clinical relevance requires further investigation, particularly in relation to hard cardiovascular outcomes. Future research should further validate its clinical value by standardizing intervention protocols, expanding sample sizes, and extending follow-up durations. This study provides critical evidence to optimize diabetes management strategies and supports the integration of nurse-led models into global diabetes prevention and care systems.

## Data Availability

The original contributions presented in the study are included in the article/Supplementary material, further inquiries can be directed to the corresponding author.
