# Supplementary material for: Impact of nurse-led self-management education on type 2 diabetes: a meta-analysis
Source: Front Public Health. 2025 Aug 11;13:1622988. doi: 10.3389/fpubh.2025.1622988 (PMC12375586; doi:10.3389/fpubh.2025.1622988)
Supplement: Supplementary file 1 [file Data_Sheet_1.docx]

# Supplementary Appendix A. Full Search Strategies

## PubMed (MEDLINE)

Searched through February 28, 2025

("type 2 diabetes mellitus"[MeSH Terms] OR "type 2 diabetes"[tiab] OR T2DM[tiab] OR "non-insulin dependent diabetes"[tiab])
AND
("nurse-led"[tiab] OR "nursing intervention"[tiab] OR "nurse educator"[tiab] OR "nurse-delivered"[tiab])
AND
("self-management education"[tiab] OR DSME[tiab] OR "patient education"[MeSH Terms] OR "behavior change"[tiab] OR "diabetes self-care"[tiab])

## EMBASE (via Elsevier)

Searched through February 28, 2025

('type 2 diabetes mellitus'/exp OR 'type 2 diabetes':ti,ab OR T2DM:ti,ab OR 'non insulin dependent diabetes':ti,ab)
AND
('nursing intervention':ti,ab OR 'nurse-led':ti,ab OR 'nurse educator':ti,ab OR 'nurse delivered':ti,ab)
AND
('self management education':ti,ab OR DSME:ti,ab OR 'patient education'/exp OR 'diabetes self care':ti,ab OR 'behavior change':ti,ab)

## Web of Science (Core Collection)

Searched through February 28, 2025

TS=("type 2 diabetes" OR T2DM OR "non-insulin dependent diabetes")
AND
TS=("nurse-led" OR "nursing intervention" OR "nurse educator" OR "nurse delivered")
AND
TS=("self-management education" OR DSME OR "patient education" OR "diabetes self-care" OR "behavior change")
