# Supplementary material for: Impact of nurse-led self-management education on type 2 diabetes: a meta-analysis
Source: Front Public Health. 2025 Aug 11;13:1622988. doi: 10.3389/fpubh.2025.1622988 (PMC12375586; doi:10.3389/fpubh.2025.1622988)
Supplement: Supplementary file 2 [file Table_1.docx]

Table S1

1A: Model Sensitivity Analysis

| Outcome | Effect (Fixed-Effects Model) | Effect (Random-Effects, REML) | Effect (Random-Effects, DL) | Interpretation |
| --- | --- | --- | --- | --- |
| HbA1c (%) | MD –0.48 (–0.64 to –0.33) | MD –0.54 (–0.86 to –0.23) | MD –0.53 (–0.84 to –0.22) | Direction consistent; REML yields wider CI due to heterogeneity |
| FBG | MD –0.18 (–0.30 to –0.06) | MD –0.20 (–0.36 to –0.03) | MD –0.19 (–0.34 to –0.03) | Effect robust across models; slightly stronger under REML |
| Total Cholesterol (TC) | MD 0.06 (–0.08 to 0.19) | MD 0.06 (–0.08 to 0.19) | MD 0.06 (–0.08 to 0.20) | No difference across models; no significant effect |
| Triglycerides (TG) | MD –0.01 (–0.15 to 0.13) | MD –0.01 (–0.15 to 0.13) | MD –0.01 (–0.15 to 0.13) | No difference; not significant under any model |
| LDL | MD 0.10 (–0.04 to 0.23) | MD 0.10 (–0.04 to 0.23) | MD 0.10 (–0.04 to 0.23) | No model-dependent difference; all estimates nonsignificant |
| HDL | MD 0.27 (0.13 to 0.41) | MD 0.27 (0.14 to 0.41) | MD 0.27 (0.13 to 0.42) | Significant effect under all models; minimal CI change |
| Self-Efficacy | SMD 1.32 (0.98 to 1.66) | SMD 1.48 (1.04 to 1.92) | SMD 1.45 (1.02 to 1.88) | Large effect across models; REML yields wider CI |

1B: Sensitivity Analysis Summary

| Analysis Condition | Pooled Effect Estimate (HbA1c, MD [95% CI]) | I² (%) | Interpretation |
| --- | --- | --- | --- |
| Primary analysis (all studies) | –0.54 (–0.86 to –0.23) | 87.80% | Reference model including all 8 studies |
| Excluding high-risk RoB studies | –0.50 (–0.78 to –0.22) | 85.20% | Slightly reduced heterogeneity; consistent effect |
| Excluding small studies (<50 participants/arm) | –0.52 (–0.84 to –0.21) | 86.40% | Stable effect; robust to study size |
| Leave-one-out: max deviation | –0.57 (–0.88 to –0.25) | 84.30% | Minimal impact of any single study removal |
